# Video-Assisted Thyroidectomy for Papillary Thyroid Carcinoma

**DOI:** 10.1155/2010/148542

**Published:** 2010-09-23

**Authors:** Celestino Pio Lombardi, Marco Raffaelli, Carmela De Crea, Annamaria D'Amore, Luigi Oragano, Massimo Salvatori, Rocco Bellantone

**Affiliations:** ^1^Division of Endocrine Surgery, Department of Surgery, Università Cattolica del Sacro Cuore-Policlinico “A. Gemelli”, L.go A. Gemelli 8, 00168 Rome, Italy; ^2^Institute of Nuclear Medicine, Università Cattolica del Sacro Cuore-Policlinico “A. Gemelli”, L.go A. Gemelli 8, 00168 Rome, Italy

## Abstract

*Background*. The results of video-assisted thyroidectomy (VAT) were evaluated in a large series of patients with papillary thyroid carcinoma (PTC), especially in terms of completeness of the surgical resection and short-to-medium term recurrence. *Methods*. The medical records of all patients who underwent video-assisted thyroidectomy for PTC between June 1998 and May 2009 were reviewed. *Results*. Three hundred fifty-nine patients were included. One hundred twenty-six patients underwent concomitant central neck node removal. Final histology showed 285 pT1, 26 pT2, and 48 pT3 PTC. Lymph node metastases were found in 27 cases. Follow-up was completed in 315 patients. Mean postoperative serum thyroglobulin level off levothyroxine was 5.4 ng/mL. Post operative ultrasonography showed no residual thyroid tissue in all the patients. Mean post-operative ^131^I uptake was 1.7%. One patient developed lateral neck recurrence. No other recurrence was observed.

## 1. Introduction

Over the last decade several techniques for minimally invasive thyroid surgery have been described, including various endoscopic [1, 2] and video-assisted approaches [3–5] as well as minimal incision techniques [6, 7]. The primary aim of all these different approaches has been to improve the cosmetic results of conventional surgery [8]. Among all these techniques, video-assisted thyroidectomy (VAT) by the central access [3, 4] is one of the most diffuse worldwide and it has been adopted by several Centers, especially in Europe [9–11] and in USA [12]. 

Initial experiences published on VAT underlined the advantages of the procedure in terms of a better cosmetic result and less postoperative pain when compared with conventional surgery [13–15]. In relatively small series of patients and comparative studies, VAT has been demonstrated to be a reproducible, safe and effective technique [3, 4]. Larger multiinstitutional series have fully demonstrated its safety and efficacy in several clinical settings [9–12]. Several advantages of this technique have been clearly demonstrated: in particular less tissue trauma and less patient discomfort [13–15]. Furthermore, in one recently published paper, we demonstrated that the incidence and the severity of early voice and swallowing postthyroidectomy symptoms are significantly reduced in patients who undergo VAT compared with those who undergo conventional surgery [16].

In the recent years, VAT has been applied successfully for the treatment of small papillary thyroid carcinomas (PTC) [17–20]. Findings of initial small comparative studies have supported the supposition that VAT allows for a surgical resection similar in terms of completeness to conventional surgery [17, 18] with no additional risk of cancer cell seeding [15]. In spite of these encouraging results, some experts consider PTC a contraindication for this approach [5, 8], and some have expressed doubts about its surgical radicality. Concern has been raised over whether this procedure is oncologically safe and adaptable beyond the few medical Centers where it has been developed and optimized [21]. Even though we and others [18, 19] have demonstrated that VAT allows to obtain the same completeness of the surgical resection as conventional thyroidectomy in patients with PTC, some surgeons are still very hesitant to treat PTC by a video-assisted approach. Additionally, one of the major criticism was about the relatively low number of cases and the absence of an adequate follow-up. Recently Miccoli et al. [20] have demonstrated, after a quite long period of follow-up that minimally invasive video-assisted thyroidectomy can be safely employed in low and intermediate risk patients without negative impact on patients outcome.

Similarly, video-assisted central compartment lymph node dissection (VALD = video-assisted lymph node dissection) has been shown to be feasible with no additional risk of complications in patients with PTC [18, 22] and in RET-gene mutation carriers [23]. 

In this paper, we retrospectively evaluated the results obtained in a large series of patients who underwent VAT for PTC over a 10-year period, especially in terms of the completeness of the surgical resection and short-to-medium terms recurrence.

## 2. Materials and Methods

Between June 1998 and May 2009, 1356 patients underwent VAT at the Division of Endocrine Surgery of the Università Cattolica del Sacro Cuore, Rome, Italy. Eligibility criteria for VAT were thyroid nodules less than 35 mm on the largest diameter, an estimated thyroid volume within the normal range (less than 30 mL), and no previous conventional neck surgery and/or radiation therapy. Absolute contraindications were malignancies other than PTC and the presence of preoperatively demonstrated infiltrating tumors or lymph node metastases. All the patients who successfully underwent VAT for an histologically proven PTC were included in this study. The medical records of these patients were retrospectively reviewed.

The operative technique has been extensively described in previously published papers [9]. 

Postoperative serum calcium and phosphorus levels were measured in all the patients. Hypocalcemia was defined as a serum calcium level below 8.0 mg/dL. Laryngoscopy was performed preoperatively only in patients that had experienced voice changes and was performed postoperatively in all patients to check vocal cord motility. 

All the patients underwent postoperative suppressive levothyroxine (LT4) treatment. All patients underwent serum thyroglobulin (sTg) and anti-Tg antibody measurements under suppressive LT4 treatment and an ultrasound (US) neck scan 3 to 6 months after surgery. This was the only follow up protocol adopted for patients with pT1 PTC ≤ 1.0 cm, in the absence of lymph node metastases and multifocality. ^131^I ablation (RAI) was performed on the basis of stage and risk factors, according to the American Thyroid Association Guidelines [24]. Low-risk patients were evaluated by ^131^I diagnostic whole body scan (DxWBS), quantitative ^131^I neck uptake (RAIU) and TSH-stimulated sTg. All the high risk patients underwent RAI. Posttherapy whole body scan (TxWBS), RAIU and TSH-stimulated sTg levels were evaluated in this group of patients.

For the purpose of this paper, the completeness of the surgical resection was determined by neck ultrasound imaging, qualitative evaluation with DxWBS or TxWBS, RAIU, and TSH-stimulated sTg levels.

sTg was measured using a commercial electrochemiluminescence immunoassay (ECLIA) (Roche Diagnostic Co., Indianapolis, IN, USA) for Elecsys System 2010. The measuring range was 0.100–1000 ng/ml. RAIU was performed by administering a capsule of 3.7 MBq 131I and 24-h isotope uptake was measured by an external probe equipped with a 2 × 2 cm NaI(Tl) crystal, attached to a series 35 multichannel analyser (ACN Monogamma, L'accessorio Nucleare S.r.l., Milano, Italy). 

RAI required hospitalization and was administered 2–6 months after surgery in hypothyroidism condition (TSH levels above 30 *μ*UI/mL), obtained by withdrawing thyroid replacement hormone. Pregnancy was excluded before ^131^I administration in all women of childbearing age. The ^131^I activity was chosen on the basis of a system of “empiric adjusted doses”. By this system, for example, low activities (i.e., 1,850 GBq) were administered in patients with unifocal pT1 PTC, no lymph node metastases, and low RAIU values, while large activities (i.e., 5,550 GBq) were chosen for multifocal pT3 PTC, with lymph node metastases and high RAIU values [24, 25]. All patients were subjected to TxWBS before discharge, 2–5 days after administration of a therapeutic dose of ^131^I, to visualize any thyroid remnants or metastases. Anterior and posterior whole body images were recorded using a large field-of view GE Starcam 3200i scintillation camera (General Electric Medical System, Milwaukee, WI, USA), fitted with a high-energy parallel-hole collimator.

Patients who did not receive RAI underwent long-term follow-up at 6–12 month intervals, at which time US scan was performed and sTg levels were measured while the patients were on LT4. Patients whose TxWBS revealed no thyroid remnants or metastases were also placed on this follow-up protocol. Patients whose TxWBS revealed thyroid remnants or metastases were subjected to DxWBS, RAIU and sTg off LT4 6–10 months after RAI. The criteria for successful thyroid ablation were defined as the disappearance of any visible area of uptake in the thyroid bed, a RAIU below 1% and undetectable sTg off LT4 (TSH > 30 *μ*UI/mL) [25]. Patients with complete thyroid remnant ablation, as defined above, underwent long-term follow-up at intervals of 6–12 months with sTg measurements taken on LT4 and US neck scan. Patients with partial ablation were treated with additional therapeutic doses of ^131^I. Tumor stage was defined according to the American Joint Committee on Cancer [26].

## 3. Results

Among the 1356 patients who underwent VAT, 370 (27.3%) were demonstrated by final histological examination to have PTC, and their medical records were reviewed for this paper. Two hundred and eighty-two out of these 370 patients have been already reported in a previously published paper [19]. 

Conversion to an open procedure was required in 2 patients because of intraoperative findings of gross central neck and upper mediastinum lymph node metastases. These patients underwent total thyroidectomy and central neck dissection (plus lateral neck dissection in one of them). Final histology showed the tumors to be pT3N1b PTC in both cases.

Video-assisted thyroid resection was successfully accomplished in 368 patients. Among them, 342 (92.9%) underwent video-assisted total thyroidectomy as the initial procedure and the remaining 26 (7.0%) underwent video-assisted thyroid lobectomy. Among patients in whom a lobectomy was performed, completion thyroidectomy was carried out by conventional procedures in 3 cases, which were operated at the beginning of the experience. Final histology showed a pT1 PTC in 2 of these cases and a pT3 in the third case. Six patients had small (<5 mm) pT1 PTC and were followed up after the initial lobectomy. None of them developed distant or local recurrence. The remaining 17 patients underwent subsequent video-assisted completion thyroidectomy. In 359 patients, total thyroid resection was achieved by the video-assisted approach (single procedure video-assisted total thyroidectomy in 342 cases; video-assisted thyroid lobectomy followed by video-assisted completion thyroidectomy in 17 cases). Follow-up data from these 359 patients were analyzed in this study. There were 323 women and 36 men with a mean age of 43.4 ± 11.2 years (range: 15–79). Preoperative diagnosis was: PTC in 104 cases (29.0%), suspicious nodule in 127 cases (35.4%), follicular (indeterminate) lesion in 111 cases (30.9%), toxic multinodular goiter in the remaining 17 (4.7%). The mean maximum diameter of the lesion evaluated preoperatively by US scan was 17.3 ± 6.3 mm (range: 6–35).

A video-assisted lymph node dissection was deemed necessary in 126 patients, 94 of whom were preoperatively diagnosed with small PTC or suspicious nodules and underwent a selective central node removal of enlarged lymph nodes; the remaining 32 of these patients received a complete video-assisted central compartment lymph node dissection (CCD = Central Compartment Dissection or level VI neck node dissection). The mean number of lymph nodes removed during VALD was 6 ± 4.06 (range: 1–19). The mean number of removed nodes in the CCD subgroup was 9.2 ± 3.7 (range: 6–19). Concomitant parathyroidectomy for a parathyroid adenoma was performed in 7 patients.

Mean operative time was 63.1 ± 27.3 min for lobectomy (range: 30–150), 66.9 ± 22.6 min for total thyroidectomy (range: 30–220), and 54.4 ± 24.9 min for completion thyroidectomy (range: 30–100). Mean operative time for CCD was 17.7 ± 3.4 min (range:12–22).

Final histology revealed 285 pT1 PTC diagnoses (multifocal in 80 cases), 26 pT2 diagnoses (multifocal in 10), and 48 pT3 diagnoses (multifocal in 16). One hundred fifty six out of the 285 pT1 PTC had a maximum diameter <1 cm. Among them 94 were diagnosed preoperatively. The remaining 62 cases had an incidental diagnosis of microcarcinoma. Lymph node metastases were found in 24 of the cases in which CCD was performed, including 3 cases of micrometastases and also in three case in which selective central node dissection was performed. Reactive changes were evident in the lymph nodes removed from the other 97 patients who underwent selective central node dissections. In patients with lymph node metastases (N1a), the primary tumor was pT1 in 15 cases and pT3 in 12 cases.

Post-operative complications included 11 transient recurrent nerve palsies (1.5% of the nerves at risk), 90 transient hypocalcaemia cases (25%), 4 permanent hyperparathyroidism cases (1.1%) and 1 postoperative hematoma (0.4%). Mean postoperative stay was 3.1 ± 1 days (range: 2–7).

Complete follow-up data were available in 315 (87.7%) of the 359 patients included in the study; 246 of these had pT1 tumors, 22 had pT2 tumors, and 47 had pT3 tumors. The mean follow-up period was 21.5 ± 5.9 months (range: 5–40). In 137 of these patients (38.2%) with pT1 PTC <1.0 cm, in the absence of lymph node metastases and multifocality, follow-up evaluation included sTg measurements on LT4 and ultrasonography. sTg was undetectable (<0.1 ng/mL) and the US neck scan showed no thyroid remnants or lymph node involvement in all these cases.

For the remaining 178 patients postoperative ultrasonography showed no residual thyroid tissue; mean sTg after LT4 withdrawal was 5.4 ± 7.5 ng/mL (range 0.1–31.4); sTg was undetectable (<0.1 ng/mL) in 33 patients (18.5%); mean RAIU was 1.7 ± 2.6% (range: 0–18.2%). RAIU was <0.5% in 33 (18.5%) of the patients and <1% in 74 (41.5%) of the patients. In the 27 patients with lymph node metastases, sTg off LT4 was 4.6 ± 4.6 ng/mL (range: 0.44–13) and mean RAIU was 1.8 ± 0.8% (range: 1.2–3.4%).

Visual TxWBS evaluation demonstrated thyroid remnants and coexisting lymph node metastases in one high-risk patient. This last patient required a conventional lateral neck dissection after unsuccessful RAI two years after the initial surgery. TxWBS after the second operation showed no residual uptake.

## 4. Discussion

Among the indications for VAT, nodule size together with thyroid volume are the most important selection's limiting factors. For this reason, small suspicious or malignant nodules are, from a technical point of view, one of the best indications for VAT. However at the beginning of the experience there were some concerns about the feasibility and safety of VAT in case of malignancy. Nonetheless, after an adequate experience being achieved, this technique has now been proposed as a valid alternative to conventional surgery also for the resection of small PTC [17–20]. 

It has been supposed that manipulation and extraction of the thyroid gland through a small skin incision might increase the risk of thyroid capsule rupture, with possible cell seeding. However, we have previously demonstrated that thyroid gland manipulation does not differ between VAT and conventional surgery and that VAT does not confer additional risk of thyroid capsule rupture and cell seeding [15].

Another important concern about VAT is whether the surgical resection obtained with this approach is sufficiently complete. Previous studies suggested that the surgical radicality of VAT (as evaluated by postoperative sTg on and off LT4, ultrasound scan and RAIU) is comparable to that of conventional surgery [17–20]. The present study confirmed in a larger patient cohort that VAT allows for an adequate surgical resection in case of small PTC.

STg on LT4 suppression therapy was undetectable in most of the patients. After LT4 withdrawal (TSH > 30 UI/l), mean sTg level was low (5.4 ng/mL) and even undetectable in many patients.

Another point to take into account is the possibility to perform a central neck node clearance by the video-assisted approach. It is well know that even though up to 80% of patients with PTC could have at least microscopic metastatic spread to cervical lymph nodes, this does not seem to affect prognosis, at least in patients younger than 45 years. For this reason prophylactic neck dissection for this group of patients is not recommended [27]. We [19, 22] and others [23] have demonstrated that removal of unexpectedly enlarged central compartment lymph nodes is feasible and safe by video-assisted approach. The endoscope allows for meticulous exploration of the central compartment and quite easy identification of even slightly enlarged lymph nodes. At the same time, thanks to the magnification of the endoscope, good exposition of the operative field and of the neck structures permits careful dissection and safe preservation of the inferior laryngeal nerve and the parathyroid glands.

The mean number of lymph nodes removed in patients who underwent CCD demonstrates that it is possible to achieve satisfactory clearance of the central compartment. The incidence of central compartment node metastasis among the patients who underwent complete video-assisted CCD was comparable to the rates reported in the literature (11%–80%) [28].

Postoperative evaluation by sTg on and off LT4, US neck scan and RAIU tests confirmed that it is possible to obtain adequate surgical resection by the VAT approach, even in patients with central compartment lymph node metastases.

The results of the present study confirm our previous reports and are in agreement with a recently published paper. Indeed, Miccoli et al. [20] demonstrated that the completeness of the surgical resection is similar for both VAT and conventional surgery even in patient with minimal lymph node involvement (“intermediate risk”).

Mean follow-up time was about 2 years, with the longest follow-up of about 3 years. This could be considered the main limitations of this paper, which is probably inadequate to assess the long-term recurrence rate of PTC, considering its indolent nature.

Indeed, depending on the initial treatment and on other prognostic variables, 30% of patients with PTC will have recurrence over several decades, with about two-thirds of the recurrences occurring within the first 10 years after initial therapy [29]. In the present series, we observed only one case of lateral neck metastases which appeared after two years after the initial video-assisted thyroidectomy. This further confirm the adequacy of VAT for the treatment of small PTC.

On the basis of the previous consideration, eligibility criteria for VAT are: thyroid nodules <35 mm; thyroid volume <30 mL; no previous conventional neck surgery and/or radiation therapy and small, low- to intermediate-risk, papillary thyroid carcinomas (PTC). 

Concerns remain regarding the precise indications for lymph nodes dissection by video-assisted approach. It would be prudent to continue to perform central neck dissection only in case of enlarged central compartment lymph nodes, unexpectedly discovered during VAT for PTC or suspicious nodules, or in case of prophylactic central neck node clearance, when required. Conversion to the conventional approach is mandatory if node clearance is not as accurate as in conventional surgery. Overt lymph node involvement still remains a contraindication for video-assisted procedures.

## 5. Conclusions

In conclusion, this study confirms that VAT is feasible and safe procedure for surgical treatment of small PTC. If selection criteria are strictly followed, it seems equivalent to conventional surgery in terms of completeness of the surgical resection. VAT can be considered a valid alternative to conventional surgery also for the treatment of small PTC, in absence of any clinical or instrumental sign of local invasion or lymph node involvement.

Nonetheless, longer follow-up evaluation could further validate its oncologic results, especially in terms of recurrence rates.
